# Domain Adaptation and Adaptive Information Fusion for Object Detection on Foggy Days

**DOI:** 10.3390/s18103286

**Published:** 2018-09-30

**Authors:** Zhe Chen, Xiaofang Li, Hao Zheng, Hongmin Gao, Huibin Wang

**Affiliations:** 1 College of Computer and Information, Hohai University, Nanjing 210098, China; chenzhe@hhu.edu.cn (Z.C.); 20140065@hhu.edu.cn (H.G.); hbwang@hhu.edu.cn (H.W.); 2 Jiangsu Collaborative Innovation Center for Cultural Creativity, Changzhou 213000, China; 3 School and Computer Information and Engineering, Changzhou Institute of Technology, Changzhou 213022, China; 4 College of Information Engineering, Nanjing Xiaozhuang University, Nanjing 210017, China

**Keywords:** object detection, foggy day, haze effect, depth information

## Abstract

Foggy days pose many difficulties for outdoor camera surveillance systems. On foggy days, the optical attenuation and scattering effects of the medium significantly distort and degenerate the scene radiation, making it noisy and indistinguishable. Aiming to solve this problem, in this paper we propose a novel object detection method that has the ability to exploit the information in the color and depth domains. To prevent the error propagation problem, we clean the depth information before the training process and remove false samples from the database. A domain adaptation strategy is employed to adaptively fuse the decisions obtained in the color and depth domains. In the experiments, we evaluate the contribution of the depth information for object detection on foggy days. Moreover, the advantages of the multiple-domain adaptation strategy are experimentally demonstrated via comparison with other methods.

## 1. Introduction

Outdoor camera surveillance systems are widely used in urban areas, and play an important role in traffic management [1] and security maintenance [2]. It is necessary for these systems to operate in all weather conditions. However, foggy days pose many difficulties for vision-based systems. The attenuated scene appearance and strong noise are the two main factors that degenerate object detection results [3,4].

Many efforts have been made to acquire clear images/videos on foggy days, and excellent results have been obtained [5,6]. However, the state-of-the-art image enhancement methods cannot significantly improve the object detection performance. The reasons are twofold. First, object detection entails segmenting objects of interest from the background. Hence, the key problem for object detection on foggy days is how to identify the deviation between the object and background. This is difficult for these enhanced images, which include many textures. Second, error propagation prevents the use of any image preprocessors. The initial errors with image preprocessors will propagate to the subsequent detection process, causing errors in the final object detection results. Hence, the preprocessor-based object detection strategy is questionable in some cases.

Despite its disadvantages, the haze effect on foggy days provides a novel cue for object detection. According to the optical imaging model, the haze concentration changes with depth [7,8]. Hence, we can present the unscaled depth through haze concentration estimation, and the depth contrast between the object and background can be presented according to the point-to-point difference in haze concentration. In addition to the RGB information in the color domain, this information provides a novel feature for object detection. For haze concentration estimation on foggy days, the most efficient method is the dark channel prior model [9]. The advantage of the dark channel prior model is that it can estimate the haze concentration using monocular images. However, its drawback is that it is quite sensitive to image noise, and therefore image outliers cause serious errors in haze concentration estimation results. To solve this problem, a novel data cleaning method is used here to filter the depth data. This can ensure the correctness of the background model, but leads to inequality in the amount of data between the depth and color domains. This problem is solved by employing the domain adaptation learning strategy. Two detectors are trained separately with the color and depth information, and the final domain-adapted detection is performed by combining these two detectors. The novelty of our method is threefold:

(i) Depth-information-based object detection on foggy days. Aiming to conquer the challenges posed by foggy days, our method exploits the depth information for object detection.
(ii) Domain-adaptation-learning-based background modeling on foggy days. Our method trains the background models with the color and depth information separately, and they are jointly trained via the domain adaptation learning strategy.
(iii) Exploring depth and color features in images on foggy days. Our method explores the features in both the color and depth domains, and fuses them for object detection on foggy days.

The paper is structured as follows. In Section 2, we present state-of-the-art research for image processing and object detection on foggy days. Section 3 introduces our proposed method. The experimental results are presented in Section 4, and our conclusions are presented in Section 5.

## 2. Related Works

Most works related to object detection on foggy days involve a combination of image dehazing and object detection methods. The former is commonly used as a preprocessor for enhancing the object appearance, whereas the object–background transition is identified by the object detection postprocessor.

### 2.1. Image Processing

Aiming to remove the haze effect of images on foggy days, various image processing methods have been proposed. Generally, image processing on foggy days is achieved by transforming the atmospheric scattering model [10,11,12,13], which can be simplified as follows:

(1) $$E(d,\lambda )=E_{dt}(d,\lambda )+E_{a}(d,\lambda ),$$

where E(d,λ) is the acquired image, $E_{dt}(d,\lambda )$ is the term originating from the object radiation, $E_{a}(d,\lambda )$ is the haze term, and d and λ are the transmission distance and wavelength of the light, respectively. In this model, the key issue is the transmission distance estimation. Narasimhan et al. found that the point-to-point contrast in haze environments is related to the depth of the points. This principle is used to remove the haze and restore the original scene radiation [11]. Results obtained using this method can provide a visually desirable presentation. Schechner et al. [14] proposed a depth estimation method based on the fact that the scene depth varies with the degree of optical polarization. This method has a robust physical basis, but requires complicated polarization imaging devices. Liang et al. found that the light polarization state changes with the scene depth, and the scene contrast can be enhanced by altering the angle of light polarization [15,16]. There are also many scene depth and ambient light estimations using high-level image features. Kopf et al. introduced a three-dimensional georeferenced terrain model to estimate the real scene depth [17]. In this method, multi-source information such as the depth, texture, and geographic information were jointly used for image relighting and dehazing. Nishino et al. proposed a Bayesian probabilistic method to estimate the scene albedo and depth from a single foggy image [18]. By combining the contextual regularized L1 norm and boundary constraint, Meng proposed a method for optimally estimating the light transmission parameter [19]. Compared with the aforementioned methods, a more efficient strategy is achieved by the dark channel prior model. According to the dark channel prior, in clear images, an extremely low value of some pixels exists on at least one color channel. Hence, the dark-channel intensity in foggy images indicates the depth-dependent haze concentration [20]. The dark channel model works well in the case of parallel ambient light, whereas its performance is seriously degenerated when a skylight is included in the image. The blocking effect and flickering artifacts pose problems, as they reduce the accuracy of the depth estimation. In order to solve these problems, Li proposed a Markov random field with an intensity value prior to update the dark channel prior model [21]. Wang et al. used a constant intensity threshold to segment the skylight regions and estimate the ambient light in other regions [22]. Qing et al. proposed a mixture-of-Gaussian (MoG) model to estimate the skylight distribution [23]. Recently, Zhu et al. fused the luminance model with the dark channel prior model to remove the haze in images [24].

### 2.2. Object Detection

Owing to the appearance degeneration and haze effect on foggy days, the deviation between the object and background is seriously degenerated. To solve this problem, most existing methods—including those mentioned above—depend on a two-phase structure: an image preprocessor followed by a detection postprocessor. The advantages and disadvantages of this strategy are discussed in the Introduction (Section 1). Additionally, there are also methods based on optimal mathematical models. Oreifej proposed a three-term low-rank matrix decomposition method to decompose the image data into three components: the scene background, turbulence of the medium, and object of interest. Then, moving objects are segmented with the L1 norm [25]. Gilles adopted a geometric spatiotemporal viewpoint to solve the atmospheric turbulence problem, and developed a model that distinguishes the movement of moving objects in the case of turbulence [26].

## 3. Proposed Method

A domain adaptation strategy underlies the novel object detection method proposed in this paper [27,28]. Information in two domains—color and depth—is explored in our method. The depth information is estimated using the dark channel prior model, in which the skylight is initially removed. Moreover, we propose a data cleaning method to eliminate false depth information, ensuring the correctness of the training data.

**Figure 1:**
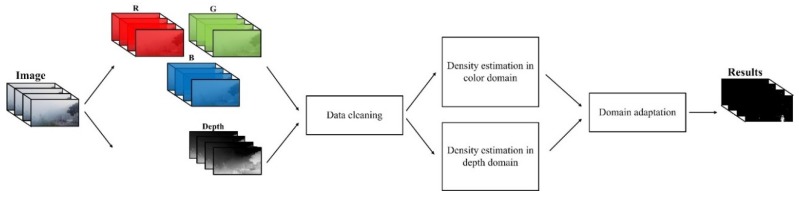
Framework of the proposed method.

After the data cleaning process, an inequality effect arises between the two sources. This problem is handled by the domain adaptation framework, and the final object detection results are generated by adaptively combining the results obtained separately using the color and depth information. The framework of our proposed method is shown in Figure 1.

### 3.1. Depth Estimate and Data Cleaning

Among existing depth estimation methods available for haze environments, the most efficient method is the dark channel prior model. Although this model allows only unscaled measurement, the estimated results can present the contrast between the object and background [22]. Aiming to remove the skylight areas, an optical feature correlation method is proposed here to recognize the light component. Moreover, to eliminate the errors in the training data, the depth information is cleaned according to the inter-frame correspondence.

#### 3.1.1. Skylight Area Recognition and Removal

In the dark channel prior model, skylight areas cause errors in depth estimation. In contrast to the ambient light transmitted through the haze medium, the intensities of all color channels of the skylight are homogeneous and significantly higher than surrounding areas [29,30]. The content of skylight areas is easily mistaken as the representation of ambient light when using the dark channel model, causing serious errors in depth estimation. The skylight can be recognized based on two aspects:

(i) Low channel variation. In contrast to other optical components, the channel variation is relatively low for the skylight.
(ii) Distance-dependent intensity. Owing to the light scattering factor in haze environments, in skylight areas, the intensity of any point is related to its distance from the optical collimation.

These two principles are mathematically modeled and combined to recognize skylight areas. For the channel variation, it can be mathematically modeled as follows:

(2) $$V_{x}^{}=\sqrt{\sum V(I_{x}^{c},I_{x}^{o})}=\sqrt{{\left(I_{x}^{r}-I_{x}^{o}\right)}^{2}+{\left(I_{x}^{g}-I_{x}^{o}\right)}^{2}+{\left(I_{x}^{b}-I_{x}^{o}\right)}^{2}},$$

where $V(I_{x}^{c},I_{x}^{o})$ is the variance of the point x in the RGB color space, $I_{x}^{c}$ is the intensity in channels (red, $I_{x}^{r}$; green, $I_{x}^{g}$; and blue, $I_{x}^{b}$), and $I_{x}^{o}$ is the average value of color channels.

For the distance-dependent intensity effect, the skylight area can be mathematically modeled using the intensity–position relation, which is scaled by an exponential distance from the highest intensity in the whole image, as follows:

(3) $${ℜ}_{x}^{}=\exp\left(D_{x,m}\right)=\exp\left(\sqrt{{\left(x_{1}-m_{1}\right)}^{2}+{\left(x_{2}-m_{2}\right)}^{2}}\right),$$

where $D_{x,m}$ is the Euclidean distance between points x and m, which have the highest intensity in the whole image. $x=\left[x_{1},x_{2}\right]$ and $m=\left[m_{1},m_{2}\right]$ are the spatial coordinates of points x and m. Combining these two principles with the correlation calculation, the discriminative function for the skylight can be modeled as follows:

(4) $$S=corr2({ℜ}_{x}^{},V_{x}^{}).$$

**Figure 2:**
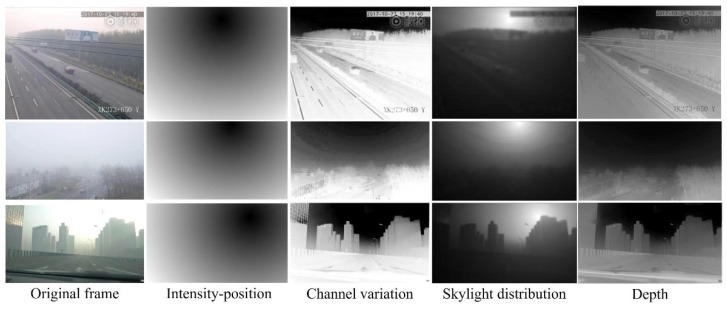
Skylight recognition and depth estimation.

The corresponding threshold for *S* is presented as *T*:

(5) $$L_{Skylight}=\left\{ \begin{array}{ll} 1 & if\;S>T\\ 0 & otherwise \end{array}\right.,$$

where corr2( ) is the two-dimensional correlation calculation, and *T* is the threshold for removing skylight areas. The ambient light estimation and the dark channel calculation are performed in the region without the skylight, where $L_{Skylight}=0$. Samples for skylight recognition and depth estimation on three foggy days are shown in Figure 2. From these samples, we can see that the correlation between the intensity position and channel variation can correctly describe the distribution of the skylight, as their values all fall into the minimum around the region of the skylight. After removing the skylight, the depth estimation results can present the sight–depth contrast between objects and the background.

#### 3.1.2. Dark Channel Prior Model-Based Depth Estimation

According to the dark channel prior model, in most of the haze-free image, an extremely low intensity value indicates at least one color channel, as follows:

(6) $$I_{x}^{dark}=\underset{y\in \Omega_{x}}{\min}(\underset{c\in \left\{r,g,b\right\}}{\min}(I_{y}^{c}))\to 0,$$

where $I_{y}^{c}$ is the channel of point y in the neighborhood and $\Omega_{x}$ is the local patch centered at point x. The intensity in the dark channel is a representation of the depth-dependent haze concentration, which is called the transmission in [20]. Hence, depth-dependent haze concentration/transmission can be expressed as follows:

(7) $$\Gamma_{x}^{}=1-\underset{y\in \Omega_{x}}{w\min}(\underset{c\in \left\{r,g,b\right\}}{\min}\left(\frac{I_{y}^{c}}{A_{}^{c}}\right)),$$

where *w* is a coefficient describing the degree of dehazing to present the depth and $A_{}^{c}$ is the ambient light that corresponds to the largest value of the dark channel over the entire images, as follows:

(8) $$A_{}^{c}=\underset{x}{\max}(I_{x}^{dark=c}).$$

As previously mentioned, the transmission $\Gamma_{x}^{}$ strictly depends on the depth $d_{x}^{}$ at point x; thus, the point-to-point depth difference can be correctly presented by the point-to-point transmission difference. Identifying the deviation between objects and the background is a desired property for object detection. Hence, in this paper, we present the depth $d_{x}^{}$ with the transmission $\Gamma_{x}^{}$, as $d_{x}^{}\propto \Gamma_{x}^{}$.

#### 3.1.3. Data Cleaning for Depth Information

**Figure 3:**
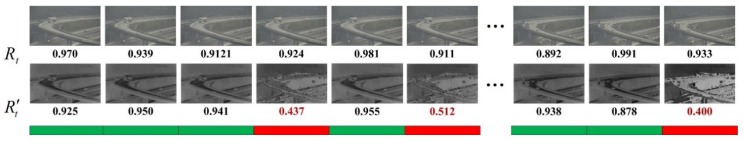
Cleaning of depth information.

Although skylight areas can be removed by the process shown in Section 3.1.1, random errors in the depth estimation—possibly caused by burr points—cannot be avoided. To solve this problem, we propose a data cleaning method for depth maps. In a video sequence, the variations between frames in a short interval are minor, and changes appear only in limited patches, whereas most pixels remain the same, as shown in the first row of Figure 3. This indicates that the correlations between frames in a short time interval are strong, which should be the case for corresponding depth maps—otherwise, random errors occur, as shown in the second row of Figure 3.

A pair of correlations between frames in a short interval and between depth maps can be mathematically calculated as follows:

(9) $$R_{t}=corr2(I_{t},I_{t-k}),$$

(10) $${R^{\prime }}_{t}=corr2(\psi_{t},\psi_{t-k}),$$

where $I_{t}$ and $I_{t-k}$ are the frames in time steps t and t−k, and $\psi_{t}$ and $\psi_{t-k}$ are the corresponding depth maps. Here, the parameter k is designed such that, for depth map $\psi_{t}$ in time step t, $\psi_{t-k}$ is the temporally nearest depth map that is correct in previous time steps. For instance, if $\psi_{t-1}$ is identified as an error in time step t−1 while $\psi_{t-2}$ is correct, k=2, $R_{t}=corr2(I_{t},I_{t-2})$, and ${R^{\prime }}_{t}=corr2(\psi_{t},\psi_{t-2})$.

Assuming that the depth map for the first frame is correct, an error is recognized under the following condition:

(11) $${R^{\prime }}_{t}<\lambda R_{t},$$

where λ is the moderation parameter. As previously mentioned, this type of error is caused by random noise, such as burr points. Thus, errors do not continually happen in a long time interval. As a result, in practice, the parameter k is not large (typically 1≤k≤3) and the temporal interval for the correlation calculation is limited, which maintains the correspondence between $R_{t}$ and ${R^{\prime }}_{t}$ for correct samples. This data cleaning process can be illustrated using samples in Figure 3. In theory, during the ambient light estimation, the dark channel prior model extracts the brightest point in the dark channel to present its neighborhood, and the brightest patch in the whole image is extracted to present the ambient light [20]. This strategy is sensitive to image burrs, as even a single noisy spot can cause errors in the ambient light estimation and depth estimation. This is a common case in real-world practice due to imaging noises. For example, the random noises located around [257, 129] in the fourth frame (first row in Figure 3) are mistaken as the representation of the ambient light due to their large intensity in the dark channel. This causes errors in depth estimation (fourth frame in the second row). As the result, the depth estimation output of the fourth frame in Figure 3 is significantly different from the previous frames, although their inputs appear to be similar. This is the reason for the low value of the ${R^{\prime }}_{t}$ obtained by the fourth frame. According to our proposed data cleaning principle, the depth estimation result of the fourth frame is eliminated, and the fifth sample is compared with the third one to continue the cleaning process.

### 3.2. Domain Adaptation Learning and Fusion Method

Taking advantage of the depth estimation method, for any scene on foggy days, two sources for object detection can be separately obtained in the color and depth domains. After data cleaning, the amount of data in these two domains is unequal. This problem is solved by a cross-source domain adaptation method, which can improve the background model with little training data (depth information) by using another model with more training data (color information). This is based on the principle that the changes in the depth scale correspond to the variations in the color information, as the depth deviation likely exists at edges where contrasts occur in the color domain. We employ the kernel density estimation (KDE) to establish the background models in the color and depth domains [31].

#### 3.2.1. KDE Model

The reason for using the KDE to establish the background models lies in its good adaptability to short-term changes of complicated scenes. Theoretically, the KDE is a typical nonparametric model that presents the background by training samples rather than any previous assumption regarding the data distribution.

The classic KDE model can be mathematically formulated by comparing the testing samples with the selected training samples, as follows:

(12) $$P(x_{t}|B_{t})=\frac{1}{N}\sum_{i=1}^{N}K(x_{t}-x_{i}),$$

where *N* samples *x_i_* are selected in the training data used as the representation of the background, $x_{t}$ represents the testing samples, *B_t_* is the background model at the time step *t*, and *K*(*x*) is the kernel function, which satisfies the conditions ∫K(x)dx=1, ∫xK(x)dx=0, and *K*(*x*) > 0.

#### 3.2.2. Color–Depth Cross-Source Domain Adaptation

The amounts of color images and depth maps are represented as $\kappa^{c}$ and $\kappa^{d}$. After data cleaning, $\kappa^{c}\geq \kappa^{d}$. We define the color as the source domain and the depth as the target domain. In these two domains, we calculate the histogram of oriented gradients (HOG) to describe the local change [32]. The HOG feature counts the number of occurrences of gradient orientation in the local regions of an image. This gradient information is useful for object detection because it changes significantly at the transition between objects and the background. For the HOG feature, every pixel has two characteristics: magnitude G and direction θ. These characteristics can be numerically presented using the LL sub-band ($\phi_{LL}$) of the discrete wavelet transform (DWT).

(13) $$G=\sqrt{\phi_{LL}^{2}(x)+\phi_{LL}^{2}(y)}$$

and

(14) $$\theta =\tan^{-1}\frac{\phi_{LL}^{}(y)}{\phi_{LL}^{}(x)},$$

where $\phi_{LL}^{}(x)$ and $\phi_{LL}^{}(y)$ are the derivatives of the LL sub-band in the *x* and *y* directions, respectively. A straightforward method for establishing the object detection feature is combining the HOG features in different domains. However, this idea is likely not appropriate for this study, owing to the inequality between the features in the source domain and the target domain.

Aiming to solve this problem, we introduce a domain adaptation learning strategy based on two parallel streams [27]. This method exploits the ample availability of training data from the color domain to learn a model that works effectively in the depth domain, for which fewer examples are available. Specifically, two separate background models $B_{t}^{c}$ and $B_{t}^{d}$ at time step *t* are trained for each domain. Let $P(x_{t}^{c}|B_{t}^{c})$ and $P(x_{t}^{d}|B_{t}^{d})$ be the distribution of the color and depth features, respectively. We can see that the distributions of the input features differ in the two domains, that is, $P(B_{t}^{c})\neq P(B_{t}^{d})$. Note that without the adaptation mechanism, this may lead to a poor detection result in the target domain, as a model in the color domain which includes a larger source training set will be trained to perform well in the dense source regions.

We now present the specific domain adaptation KDE algorithms. One of the simplest possible strategies for domain adaptation consists of a convex combination of two KDEs learned independently from the color and depth domains. Despite its simplicity, this framework has been demonstrated to yield good empirical results. As a result, the final domain-adapted background model $B_{t}^{}$ can be generated by a weighted linear combination of two background models in two different domains, as follows:

(15) $$\begin{array}{ll} P(x_{t}|B_{t}) & =w^{c}P(x_{t}^{c}|B_{t}^{c})+w^{d}P(x_{t}^{d}|B_{t}^{d})\\ & =w^{c}\frac{1}{N^{c}}\sum_{j=1}^{N^{c}}K(x_{t}^{c}-x_{j}^{c})+w^{d}\frac{1}{N^{d}}\sum_{k=1}^{N^{d}}K(x_{t}^{d}-x_{k}^{d}) \end{array}$$

where the weight parameters $w^{c}$ and $w^{d}$ are determined by minimizing the detection errors on the target (depth) domain; $w^{c}+w^{d}=1$, $x_{t}^{c}$, and $x_{t}^{d}$ are the testing samples at time step *t*; $x_{j}^{c}$ and $x_{k}^{d}$ are the background samples learned by the KDE model; and $N^{c}$ and $N^{d}$ are the numbers of background samples.

The parameters $w^{c}$ ∈ [0, 1], $w^{d}$ ∈ [0, 1] are determined via grid search by minimizing multiclass errors on the color training set. We avoid biased estimates resulting from learning the hypothesis *B_t_*, $w^{c}$, and $w^{d}$ on the same training set by applying a two-stage procedure. First, we learn distinct hypotheses using cross-validation (with the hyperparameter value found for KDE) and compute a prediction at each training sample using the cross-validation hypothesis that is not trained on that example. Second, we use these predicted outputs to determine the optimal weights. Finally, we learn the background model using the entire target training set. In general, our depth–color feature learning and fusion process can be expressed in Algorithm 1.

**Algorithm 1.** Color–Depth Domain Adaptation Learning**Input**: color images $x_{}^{c}$ (source domain), depth maps $x_{}^{d}$ (target domain)**Output**: optimal weight parameters $w^{c}$ and $w^{d}$**Initialization**: weight parameters $w^{c}=w^{d}=0.5$
Extract the HOG features in source and target domains // Equations (13) and (14)Establish the background models separately in the source domain $P(x_{t}^{c}|B_{t}^{c})$ and target domain $P(x_{t}^{d}|B_{t}^{d})$ // Equation (12)Calculate the combined detection result $P(x_{t}|B_{t})$ // Equation (15)Optimize the weight parameters $w^{c}$ and $w^{d}$ by minimizing the detection error on the target (depth) domain


## 4. Experimental Results

For experimental evaluation of our method, we selected public videos from YouTube that include diverse scenes from foggy days [33,34,35,36]. Fifty video sequences acquired on foggy days were included in this evaluation. For each sequence, we collected only one video slice, maintaining the diversity of the testing data. Hence, 50 diverse video slices with 1257 frames were tested in our experiment. For a video slice, the variations between consecutive frames were extremely low. As a result, the redundancy was high in the training dataset. If the background model was trained with all the frames, the training process would be extremely high in time cost. Aiming to remove the redundancy between training samples, we randomly selected one frame within every five-frame interval. In each of the experiments, we kept the resolution of the input frames as the original resolution of the frames. For a video sequence, we selected 250 frames as training samples to model the background. First, we demonstrate the contribution of the depth information by presenting the object detection results with and without depth information. Then, our method is experimentally compared with the existing background modeling methods, that is, spatiotemporal MoG (ST-MoG) [37], Vibe [38], and DECOLOR [39]. Moreover, the phase spectrum of the quaternion Fourier transform (PQFT) method was also selected as a typical preprocessor-/saliency-based object detection method [40]. For these compared methods, features extracted in the color domain were employed, and depth features were not included. Thus, the performance of the domain adaptation strategy can be fairly demonstrated. The excellent deep learning methods were not included in our experiments because they require a large amount of training data, exceeding that collected in this study. Currently, there is no database that contains enough data acquired on foggy days for training a deep network. If we implemented the deep learning methods with databases similar to that of the KDE models, it would be difficult to achieve the desired object detection results, yielding an unfair evaluation of these deep learning methods. Hence, the methods selected for comparison in this study are relatively low in model complexity and have been proven to be efficient for object detection in diverse scenes. The parameters T and λ were set as T=0.8 and λ=0.9, and the window size of the dark channel prior was 3 × 3 in our experiment.

### 4.1. Evaluation Criterion

The ground truth in our experimental evaluations was obtained by the average of the labels provided by 10 volunteers. Because the purpose of our method is to detect regions of moving objects, moving objects in our ground truth were identified according to a hypothesis: moving objects are identified if their displacement is larger than 10 pixels in 5 consecutive frames. This can prevent the influence of static objects and dynamic noise. According to the PASCAL criterion [41], *C* was used to evaluate the overlap of the detection results and the ground truth:

(16) $$C=\frac{\Omega^{\prime }\cap \Omega }{\Omega^{\prime }\cup \Omega },$$

where $\Omega^{\prime }$ represents the detected results and Ω represents the ground truth. The performance of our method was evaluated with respect to six criteria [42]: precision (Pr), similarity (Sim), true positive rate (TPR), F-score (FS), false positive rate (FPR), and percentage of wrong classifications (PWC), as follows:

(17) $$\mathrm{Pr}=\frac{tp}{tp+ft},\;\mathrm{TPR}=\frac{tp}{tp+fn},\;\mathrm{Fs}=2\times \frac{\mathrm{Pr}\times \mathrm{TPR}}{\mathrm{Pr}+\mathrm{TPR}},\;\mathrm{Sim}=\frac{tp}{tp+fp+fn},\;\mathrm{FPR}=\frac{fp}{fp+tn},\;\mathrm{PWC}=100\times \frac{fn+fp}{tp+tn+fp+fn}$$

Here, *tp*, *tn*, *fp*, and *fn* denote the numbers of true positives, true negatives, false positives, and false negatives, respectively.

### 4.2. Qualitative Evaluation

The performance in two aspects is demonstrated here. First, the object detection performance using the depth information is presented, from which we can qualitatively understand the motivation for combining the information in the color and depth domains. Second, the performance is qualitatively evaluated via comparison with other methods.

**Figure 4:**
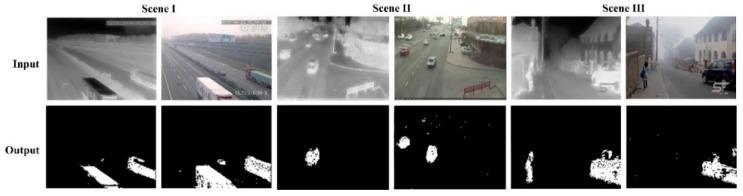
Object detection results obtained using the depth (gray) and color (RGB) information. For each scene, the right panel is the result given by the depth information and the left given by the color information.

Figure 4 shows maps of the depth, color information, and corresponding object detection results for three scenes on foggy days. We observed a complementary relationship between the depth and color information. Generally, the depth information was more sensitive to nearby objects and had a better capability to remove the background noise. However, it was difficult to detect distant objects using the depth information. In contrast to the depth information, the color information performed better for detecting objects far from the camera. However, there were point-like noises in the results obtained with the color information. As a result, we observed that some of the distant objects in Scenes I and II were missed when the depth information was used. However, the depth information performed better for Scene III, as it identified the pedestrian object that was missed in the results obtained using the color information.

**Figure 5:**
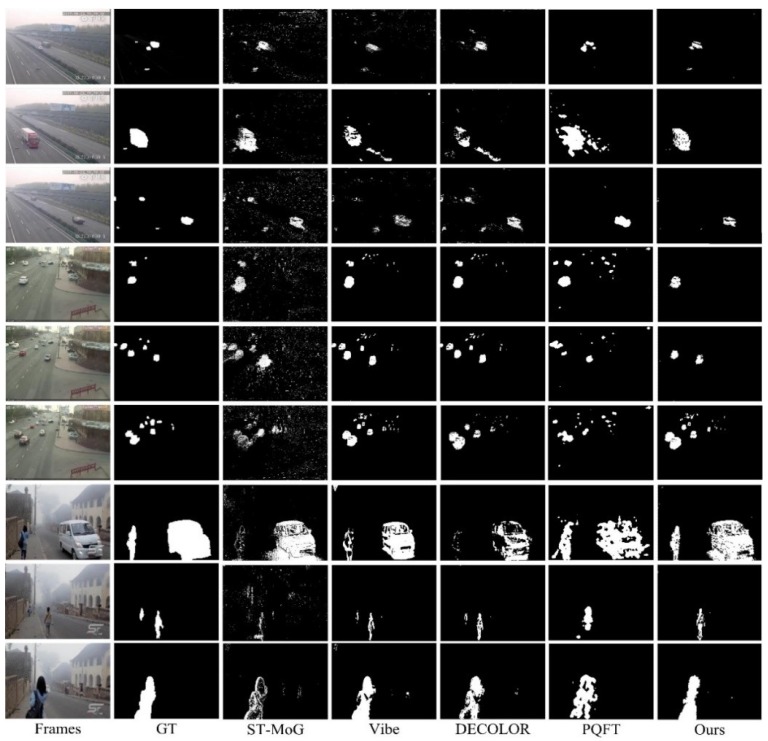
Performance comparison. GT: ground truth; PQFT: phase spectrum of the quaternion Fourier transform; ST-MoG: spatiotemporal mixture-of-Gaussian.

A qualitative performance comparison is shown in Figure 5. These results indicate diverse properties among the different methods. The ST-MoG model had a good ability to identify objects, but the drawback of this method can be demonstrated by the noisy points, as well as by the holes in the results. The Vibe and DECOLOR methods presented a better performance for block-like objects. However, the performance of DECOLOR was relatively degenerated when structural objects were close to the camera, such as the samples in the seventh and last rows. The PQFT-based method could only provide rough results, depicting the object areas rather than their accurate contours. Generally, the best results were provided by the proposed method, as it could correctly detect objects, especially nearby ones. However, our method missed distant objects in some cases (e.g., the results in the third and fourth rows). The reason for this error is that the depth information used in our approach is given by the simple dark channel prior model-based unscaled measurement, which has high efficiency but low resolution. Hence, the objects that were far from the camera were likely mistaken as the background, as the unscaled depth information is not sensitive enough to distinguish movements occurring far from the camera. Morphological schemes such as the erosion and dilation operator [43] can be further introduced here to remove the burrs and noise in the results. However, these methods cannot run automatically, and we should carefully configure the controlling parameters, such as the bandwidth of the kernel, on a case-by-case basis. This may lead to a bias for performance comparisons because we can hardly determine whether these parameters are globally optimal for any special results. This is the reason for maintaining the original object detection results in the experimental evaluations.

### 4.3. Quantitative Evaluation

Using the aforementioned criteria, we provide a quantitative evaluation of the compared methods and our method. As indicated by Table 1, our method exhibited the best performance for four criteria and the second-best performance for two criteria. The most comparable performance was achieved by the Vibe method, as it was the best for two criteria and provided the second-best results for three criteria. According to the scores in Table 1, although the performance of our method could not reach the performance achieved in good weather, our method could be applied in most cases, as an average score of the PASCAL criterion $\bar{C}\;>\;0.5$ indicates successful detection and tracking.

## 5. Conclusions

In order to solve problems with object detection on foggy days, this study explores and fuses the color and depth information from image data. A series of tricks, such as skylight removal and data cleaning, are proposed to prevent errors in the training dataset. We separately trained and established background models using the features from the color and depth domains. These two background models were combined under a unified domain adaptation framework, which introduces the model in the source domain (color) to the target domain (depth). In our experiments using public data on foggy days, we achieved the desired object detection results. A potential disadvantage of our method, as indicated by the experimental results, is difficulty in detecting distant objects. This problem can probably be solved by updating the depth estimation method.

Our method is the first to investigate depth-feature-based object detection on foggy days. The strategy of our method can be generalized to other object detection tasks where depth information is available, such as RGB-D data-based object detection. Moreover, a fog detection model is included in our future work, which underlies an all-weather system in real-life conditions.

## Figures and Tables

**Table 1:** Quantitative performance comparison of ST-MoG, Vibe, DECOLOR, PQFT, and our method. FPR: false positive rate; FS: F-score; Pr: precision; PWC: percentage of wrong classifications; Sim: similarity TPR: true positive rate.

Method	C¯	Pr	TPR	FS	Sim	FPR	PWC
ST-MoG	0.4015	0.4686	0.4826	0.5341	0.5365	0.1062	3.8571
Vibe	0.5951	0.7690	0.5952	0.4298	0.6001	0.0551	4.1294
DECOLOR	0.5254	0.4518	0.5719	0.5526	0.5636	0.0687	2.7658
PQFT	0.4217	0.5041	0.4124	0.4217	0.4614	0.1547	6.3871
Our method	0.6147	0.6215	0.6754	0.5955	0.5997	0.0425	3.9200
